# Immune ULBP1 is Elevated in Colon Adenocarcinoma and Predicts Prognosis

**DOI:** 10.3389/fgene.2022.762514

**Published:** 2022-02-08

**Authors:** Guo-Tian Ruan, Hai-Lun Xie, Li-Chen Zhu, Yi-Zhong Ge, Lin Yan, Cun Liao, Yi-Zhen Gong, Han-Ping Shi

**Affiliations:** ^1^ Department of Gastrointestinal Surgery/Department of Clinical Nutrition, Beijing Shijitan Hospital, Capital Medical University, Beijing, China; ^2^ Beijing International Science and Technology Cooperation Base for Cancer Metabolism and Nutrition, Beijing, China; ^3^ Department of Immunology, School of Preclinical Medicine, Guangxi Medical University, Nanning, China; ^4^ Department of Thoracic Surgery, Affiliated Hospital of Guilin Medical College, Guilin, China; ^5^ Department of Colorectal and Anal Surgery, The First Affiliated Hospital of Guangxi Medical University, Nanning, China; ^6^ Division of Colorectal and Anal Surgery, Department of Gastrointestinal Surgery, Guangxi Medical University Cancer Hospital, Nanning, China

**Keywords:** ULBP1, COAD, NKG2D, GSEA, biomarker (BM)

## Abstract

**Background:** Colon adenocarcinoma (COAD) is still the main cause of cancer deaths worldwide. Although immunotherapy has made progress in recent years, there is still a need to improve diagnosis, prognosis, and treatment tools. UL-16 binding protein 1 (ULBP1) is a ligand that activates the receptor natural killer cell group 2 receptor D (NKG2D) and plays an important immunomodulatory role. We aimed to investigate the clinical significance of *ULBP1* in COAD.

**Methods:** We obtained the relevant data from The Cancer Genome Atlas (TCGA). A total of 438 patients with COAD were included in this study, with a mean age of 67.1 ± 13.03 years old, of which 234 (53.42%) were male. The diagnostic value of COAD tumor tissues and adjacent tissues was analyzed by ROC curve. Univariate and multivariate survival analysis investigated the prognostic value of *ULBP1* gene, and Gene Set Enrichment Analysis (GSEA) curve was performed to analyze the biological process and enriched enrichment pathway of *ULBP1* in COAD. Combination survival analysis investigated the combined prognostic effect of prognostic genes.

**Results:** *ULBP1* gene had a high diagnostic value in COAD [AUC (TCGA) = 0.959; AUC (Guangxi) = 0.898]. Up-regulated *ULBP1* gene of patients with COAD predicted a worse prognosis compared to those patients with down-regulated *ULBP1* gene (Adjusted HR = 1.544, 95% CI = 1.020–2.337, *p* = 0.040). The GSEA showed that *ULBP1* was involved in the apoptotic pathway and biological process of T cell mediated cytotoxicity, regulation of natural killer cell activation, and T cell mediated immunity of COAD. The combination survival analysis showed that the combination of high expression of *ULBP1*, *AARS1*, and *DDIT3* would increase the 2.2-fold death risk of COAD when compared with those of low expression genes.

**Conclusion:** The immune-related *ULBP1* gene had diagnostic and prognostic value in COAD. The combination of *ULBP1*, *AARS1*, and *DDIT3* genes could improve the prognostic prediction performance in COAD.

## Introduction

According to Global Sung et al. (2021), it is estimated that there will be more than 1.9 million new cases of colorectal cancer (CRC) and 935,000 deaths, accounting for about one-tenth of cancer cases and deaths. Overall, the incidence of CRC ranks third, but the mortality rate ranks second (Sung et al., 2021). Colon adenocarcinoma (COAD) is a common malignant tumor of the digestive system, and it is the most frequently diagnosed histological subtype of CRC (Siegel et al., 2020). The patient’s clinical symptoms usually manifest as diarrhea, abdominal pain, and bloody stools, which develop in the middle and late stages of the disease. The quality of life of patients is usually very low, and the prognosis of most patients is poor. The occurrence and development of COAD are the results of a variety of mixed factors *in vivo* and *in vitro*, which involve a series of molecules and signal pathways (Wang et al., 2021). Although substantive diagnosis and treatment strategies such as surgery, neoadjuvant therapy, and targeted therapy are constantly being developed, the recurrence rate of postoperative COAD is still high, and the 5-year survival rate of patients with advanced COAD is still very low (The 5-year survival rate after distant metastasis is less than 15%) (Patel et al., 2021). Thus, there is an urgent need to explore new biomarkers and therapeutic targets in clinical practice to improve the survival rate of patients with COAD.

Natural killer cell group 2 receptor D (NKG2D) is an alkaline-activated receptor belonging to the c-type lectin-like family. It is expressed in NK cells, most NKT cells, some *γδ* T+, and CD8 T+ cells. It is different from other NKG2 receptors, which is not associated with CD94 (Mondelli, 2012) and has nothing to do with CD94 (Mondelli, 2012). The seemingly unchanged activation receptor NKG2D is mixed with a variety of ligands, such as the major histocompatibility complex class I-related chain A and B (MICA and MICB) and the unique long 16 (UL16)-binding protein family (ULBPs, ULBP1-6) which are poor (Champsaur and Lanier, 2010). NKG2D ligand expression is usually lacking in healthy tissue but can induce expression under stress, infection, and DNA damage. NKG2D ligand is also widely expressed in a variety of cancer cell lines and primary solid tumors (McGilvray et al., 2009; Champsaur and Lanier, 2010). The upregulation of these ligands may break NK cells from inhibiting the balance of activation (induced self-identification), with significant biological significance. The interaction of NKG2D is variable between different types of cancers. In the mouse model, the tumor cell line of transfection of RaE1 is rejected by NKG2D-mediated immunization (Diefenbach et al., 2001). The most recent NKG2D knockout mice provide the most convincing evidence for NKG2D to participate in anti-tumor immune responses (Guerra et al., 2008). Many mechanisms have been proposed, cancer can evade NKG2D-mediated immune response. In some systems, the persistent expression of NKG2D ligands can cause NKG2D expression to be lowered (Oppenheim et al., 2005). These results indicate that NKG2D’s participation in the anti-cancer immune response is significantly different between different types of cancer. It is also proposed that tumors may release soluble NKG2D ligands, or secrete immunosuppressive cytokines, such as transforming growth factor-beta to reduce NKG2D expression (Groh et al., 2002; Castriconi et al., 2003; Lee et al., 2004). Notably, NKG2D ligands can be independently expressed in human cell lines and primary tumors, the expression of NKG2D ligands among different tumors in knockout mice is also heterogeneous (McGilvray et al., 2009). The complex interaction between NKG2D and its ligands may involve the natural history and treatment response of various cancers (Mondelli, 2012).

The authors showed that *ULBP1*, one of the important ligands of NKG2D, is up-regulated in COAD cancer tissues, but is low-expressed in normal adjacent tissues. Although most previous studies reported that *ULBP1* was related to recurrence-free survival, disease-free survival, or overall survival (OS) in different cancers (McGilvray et al., 2009; McGilvray et al., 2010; Mondelli, 2012; Chen et al., 2013; Maccalli et al., 2017), the relationship between *ULBP1* and OS in COAD has not been reported yet. Therefore, our study uncovers and investigates the diagnosis, prognosis, and immune mechanism of *ULBP1* gene in COAD, which may help make this immune receptor an exceptional candidate for basic and applied cancer research in COAD.

## Materials and Methods

### Public Data Collection

We downloaded the COAD-related *ULBP1* gene mRNA expression data set and the corresponding patient clinical information parameters from the public cancer database-The Cancer Genome Atlas (TCGA, https://tcga-data.nci.nih.gov/, obtained on December 10, 2020) (Giordano, 2014; Hutter and Zenklusen, 2018). Based on the TCGA-COAD project data, the differential expression level of *ULBP1* in tumor tissues and adjacent normal tissues in pan-cancers was obtained from the TIMER website (https://cistrome.shinyapps.io/timer/, obtained on June 10, 2021) (Liu et al., 2021). The expression level of *ULBP1* gene in COAD tumor tissues and adjacent normal tissues was also obtained from the GEPIA website, which integrated the expression levels of normal tissues in the TCGA database and the Genotype-Tissue Expression (GTEx) database. Additionally, the expression level of *ULBP1* in different COAD tumor stages was obtained from Gene Expression Profiling Interactive Analysis (GEPIA; http://gepia.cancer-pku.cn/index.html; obtained on June 10, 2021) (Tang et al., 2017). We obtained the methylation level and mutation status of *ULBP1* gene from UALCAN database (http://ualcan.path.uab.edu/index.html, obtained on June 10, 2021) (Li et al., 2021) and cBio Cancer Genomics Portal (cBioPortal, https://www.cbioportal.org/, obtained on June 12, 2021), respectively (Harbig et al., 2021). The ULBP1-expressed protein in COAD cancer tissues and adjacent normal tissues was obtained from THE HUMAN PROTEIN ATLAS (HPA, https://www.proteinatlas.org/, obtained on June 12, 2021) (Ullah et al., 2021). Finally, we obtained the information on immune infiltration associated with *ULBP1* in COAD and the correlation between *ULBP1* gene expression level and Genomics of Drug Sensitivity in Cancer (GDSC) drug sensitivity test or The Cancer Therapeutics Response Portal (CTRP) drug sensitivity test in pan-cancer from the Gene Set Cancer Analysis (GSCA, https://www.proteinatlas.org/, obtained on June 13, 2021) (Ji et al., 2016).

### Validation of the Differential Expression and Diagnostic Value of UL-16 Binding Protein 1

COAD tumor tissues and adjacent normal tissues were obtained from the Department of Colorectal Surgery, First Affiliated Hospital of Guangxi Medical University. Inclusion criteria included: 1. The age is not less than 18 years old; 2. The postoperative pathological diagnosis is COAD; 3. Sign the surgical consent form and informed consent form; 4. The length of the hospital stay is more than 48 h. Exclusion criteria included: 1. Suffer from multiple tumors at the same time; 2. Has received preoperative neoadjuvant radiotherapy and chemotherapy; 3. Refuse to sign the informed consent form; 4. The age is less than 18 years old; 5. The length of hospitalization is less than 48 h. After the tissue was excised, it was cut into RNA protection solution and quickly stored in the refrigerator at −80°C. The total RNA extracted from the tissue was reverse transcribed into cDNA and then the qPCR reaction program was performed. The PCR reaction program was performed according to the following conditions: 95°C for 10 min, 1 cycle; 95°C for 15 s, 60°C for 1 min, and 95°C for 30 s, 40 cycles; 95°C for 15 s, 60°C 1 min, 95°C for 30 s, 60°C for 15 s, 1 cycle. Use *GAPDH* as a reference gene: upstream, 5′-GTC​AGC​CGC​ATC​TTC​TTT-3'; downstream, 5′-CGC​CCA​ATA​CGA​CCA​AAT-3'. The target *ULBP1* gene sequence was: upstream, 5′-CAC​ACA​CTG​TCT​TTG​CTA​TGA​C-3'; downstream, 5′- CCA​GGT​TTT​TGT​GAC​ATT​GAC​T-3'. The relative expression level of *ULBP1* gene was performed according to previous descript method of 2-∆∆ Cq (Ruan et al., 2020a).

### Comprehensive Analysis of the Clinical Value of UL-16 Binding Protein 1 Gene Based on the The Cancer Genome Atlas Cohort

In the TCGA database, patients were divided into two high- and low-expression groups based on the median cut-off value of *ULBP1* gene expression. Univariate and multivariate survival analysis was performed to assess the potential prognostic value of *ULBP1* gene expression in patients with COAD.

According to the expression level of *ULBP1* gene, the COAD expression genome-wide data in the TCGA database was divided into high expression group and low expression group. When the gene expression satisfied **|**log_2_foldchange**|**≥1 and *p* < 0.05, it was considered to be a differential expression gene in this study.

We investigated the co-expression analysis of *ULBP1* and COAD-related genes in the TCGA cohort. When the Pearson correlation coefficient ≥0.3 or ≤ −0.3, and the *p*-value < 0.05, it was considered to be a co-expressed gene with the *ULBP1* in COAD. The top 20 co-expressed genes were selected to analyze the prognostic value of genes in COAD. Significant prognostic genes were selected to construct a risk score model based on the prognostic contribution coefficients (*β*) of different genes. The risk score was generated based on the calculation formula: gene expression of 1*β1+ gene expression of 2*β2+…+gene expression of n*βn (Ruan et al., 2020b).

### Statistical Analysis

In this study, the unpaired Student’s *t*-test or paired *t*-test was used to compare the expression levels between two groups. The gene expression level was expressed by using the mean ± standard deviation (SD). If the data did not conform to the normal distribution, the rank sum test was used. Univariate and multivariate cox regression analyses were performed to investigate the prognostic value of genes. The selection of adjustment variables adopted single-factor meaningful clinical parameters, and the TNM stage was used as an adjusted factor for prognostic adjustment to reduce the clinical deviation. All two-tailed *p* < 0.05 were considered statistically significant. The statistical analyses in this study were performed using SPSS 22.0 version and R platform, version 4.0.1.

## Results

### Baseline Characteristics

In this study, a total of 456 patients with *ULBP1* mRNA expression data set in COAD were obtained from the TCGA database, including 480 tumor tissue samples and 41 adjacent normal tissue samples. After removing the duplicate information and the information with a survival time of 0, we obtained a total of 438 tumor sample information and 42 adjacent normal tissue sample information. The mean age of the 438 patients was 67.1 ± 13.03 years old, including 234 males (53.42%) and 204 females (46.58%). Clinical parameter information included age, sex, and TNM stage. The univariate survival analysis of clinical parameters showed that only the TNM stage had a significant prognostic value in COAD (*p* < 0.001, Table 1).

**TABLE 1:** Baseline patient characteristics in TCGA cohort

Variables	Patients	OS			
	(*n* = 438)	No. of events	MST (days)	HR(95%CI)	Log-rank P
Age (years)					
≤65	175	29	NA	1	0.062
>65	261	68	2,134	0.064 (0.429–1.024)	—
Missing*	2	—	—	—	—
Sex					
Male	234	54	2,475	1	0.545
Female	204	44	NA	0.884 (0.593–1.318)	—
TNM stage					
I	73	4	NA	1	<0.001
II	167	27	2,821	2.240 (0.781–6.421)	—
III	126	31	NA	4.068 (1.434–11.538)	—
IV	61	31	858	11.291 (3.980–32.026)	—
Missing^#^	11	—	—	—	—

Notes: Missing*, information of age was unknown in 2 patients; Missing^#^, information of TNM stage was not reported in 10 patients; TCGA, The Cancer Genome Atlas; OS, overall survival; MST, median survival time; 95 % CI, 95 % confidence interval; HR, hazards ratio; NA, not available.

### Investigating the Association Between UL-16 Binding Protein 1 Gene Expression and Immune Infiltration and Drug Sensitivity

**FIGURE 1:**
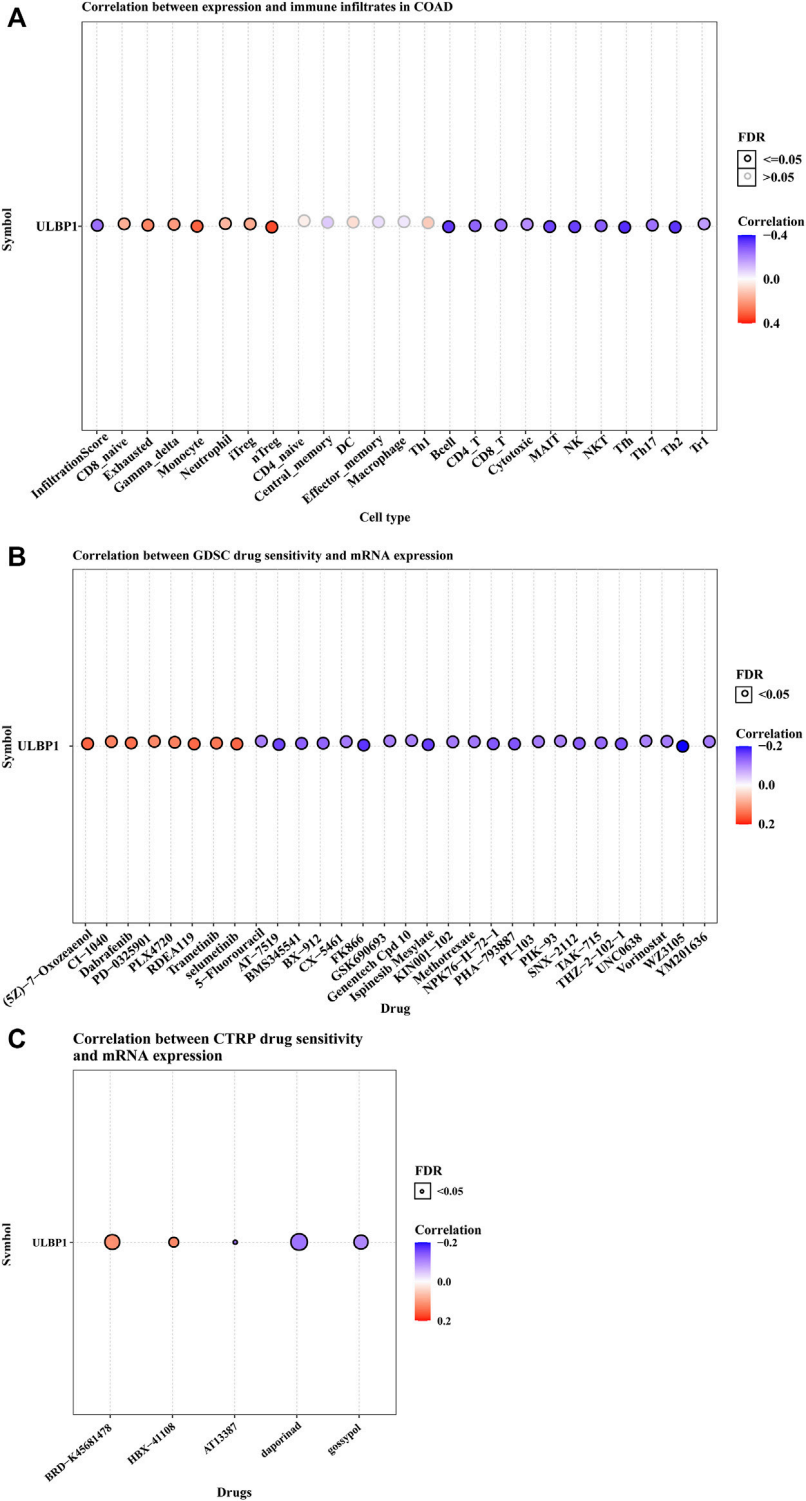
ULBP1’s immune infiltration in COAD and the relationship between ULBP1 and pan-cancer drug sensitivity tests based on GSCA. **(A)** immune infiltration; **(B,C)** GDSC and CTRP drug sensitivity test. Notes: COAD, colon adenocarcinoma; ULBP1, unique long 16 (UL16)-binding protein 1; GDSC, Genomics of Drug Sensitivity in Cancer; CTRP, The Cancer Therapeutics Response Portal; GSCA, Gene Set Cancer Analysis.

Based on the GSCA website, the association between *ULBP1* gene expression and immune infiltration suggested that the *ULBP1* expression was significantly positively related to the cells of nTreg, iTreg, Neutrophil, Monocyte, Gamma_delta, Exhausted, and CD8_navie. However, the inverse relationship was observed in the cells of NK, NKT, Tfh, Th17, Th2, Tr1, MAIT, Cytotoxic, CD8_T, CD4_T, and B cell. Additionally, a correlational relationship was observed in *ULBP1* gene expression and the majority of drug sensitivity (Figure 1).

### Differential Expression Analysis and Diagnostic ROC Curve Analysis

**FIGURE 2:**
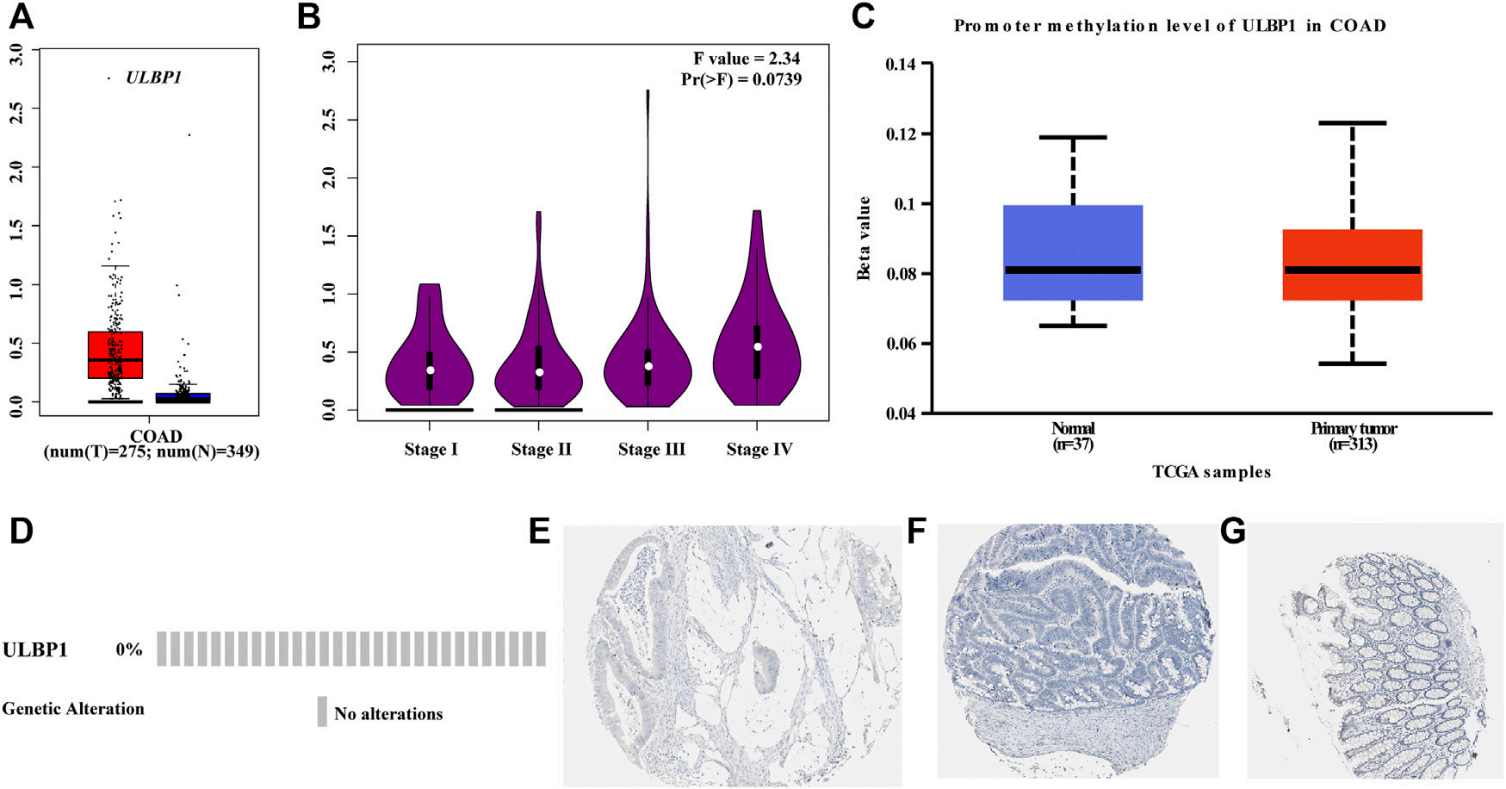
*ULBP1* and *ULBP1* methylation levels in tumor tissues and adjacent normal tissues, ULBP1 mutations, and protein expression in COAD. **(A,B)** GEPIA data: *ULBP1* expression level in COAD and different COAD tumor stages; **(C)** *ULBP1* methylation levels in COAD; **(D)** *ULBP1* mutation; **(E–G)** immunohistochemistry. Notes: COAD, colon adenocarcinoma; *ULBP1*, unique long 16 (UL16)-binding protein 1; GEPIA, Gene Expression Profiling Interactive Analysis.

**FIGURE 3:**
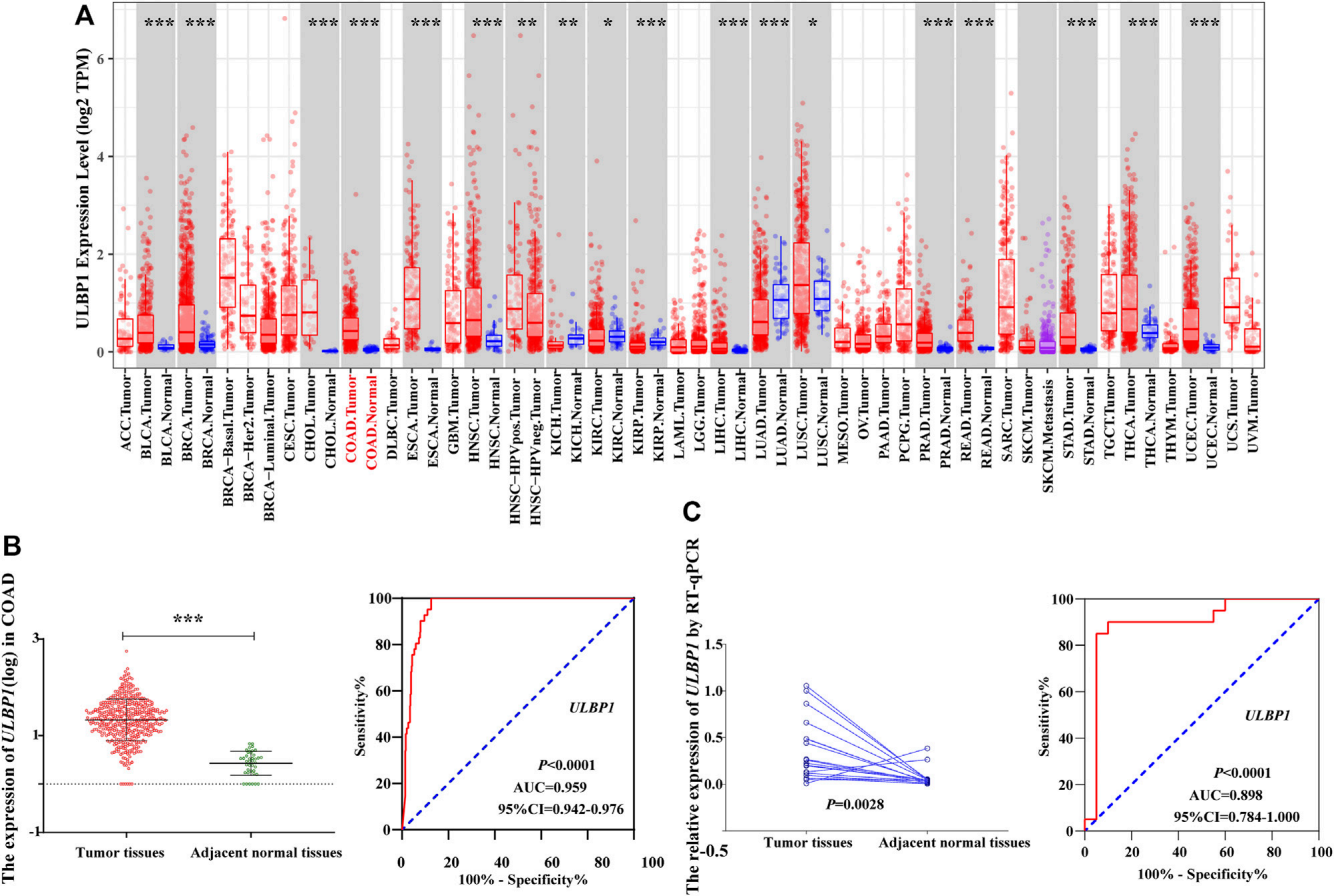
The expression level of *ULBP1* gene in pan-carcinoma and the diagnostic ROC curve. **(A)** The expression level of *ULBP1* gene in pan-cancers based on TIMER; **(B,C)** ROC curve of TCGA cohort and Guangxi validation cohort. Notes: COAD, colon adenocarcinoma; ULBP1, unique long 16 (UL16)-binding protein 1; ROC, receiver operating characteristic curve. *p < 0.05; ***p < 0.001; ****p < 0.0001.

We obtained the *ULBP1* gene in COAD tumor tissues and normal tissues from the GEPIA database that matched the information from the GTEx database and found that *ULBP1* expression was up-regulated in tumor tissues and also found that the expression level of *ULBP1* gene increased with the progression of tumor stages (Figures 2A,B). It was also found that the expression level of *ULBP1* in most tumor tissues was higher than that in normal tissues adjacent to cancer (Figure 3A). There was no significant difference in the methylation level of *ULBP1* gene in tumor tissues and adjacent normal tissues in COAD, and the mutation rate of *ULBP1* gene in COAD was 0% (Figures 2C,D). *ULBP1* expressed protein was mainly expressed in the cytoplasm (Figures 2E–G).

Based on the TCGA cohort, we analyzed the expression level and diagnostic value of *ULBP1* gene in COAD. The results showed that the expression level of *ULBP1* gene in COAD tumor tissues was significantly higher than that in adjacent normal tissues (*p* < 0.001). Simultaneously, it had a higher diagnostic value in COAD (AUC = 0.898, 95%CI = 0.784–1.000, *p* < 0.0001) (Figure 3B).

The validation result based on the Guangxi cohort found that the expression level of *ULBP1* gene in COAD tumor tissue was significantly higher than that in adjacent normal tissues (*p* = 0.0028). The diagnostic ROC curve results showed that *ULBP1* has a higher diagnostic value in COAD (AUC = 0.959, 95%CI = 0.942–0.976, *p* < 0.001) (Figure 3C).

### Survival Analysis of UL-16 Binding Protein 1 Gene in Colon Adenocarcinoma

**FIGURE 4:**
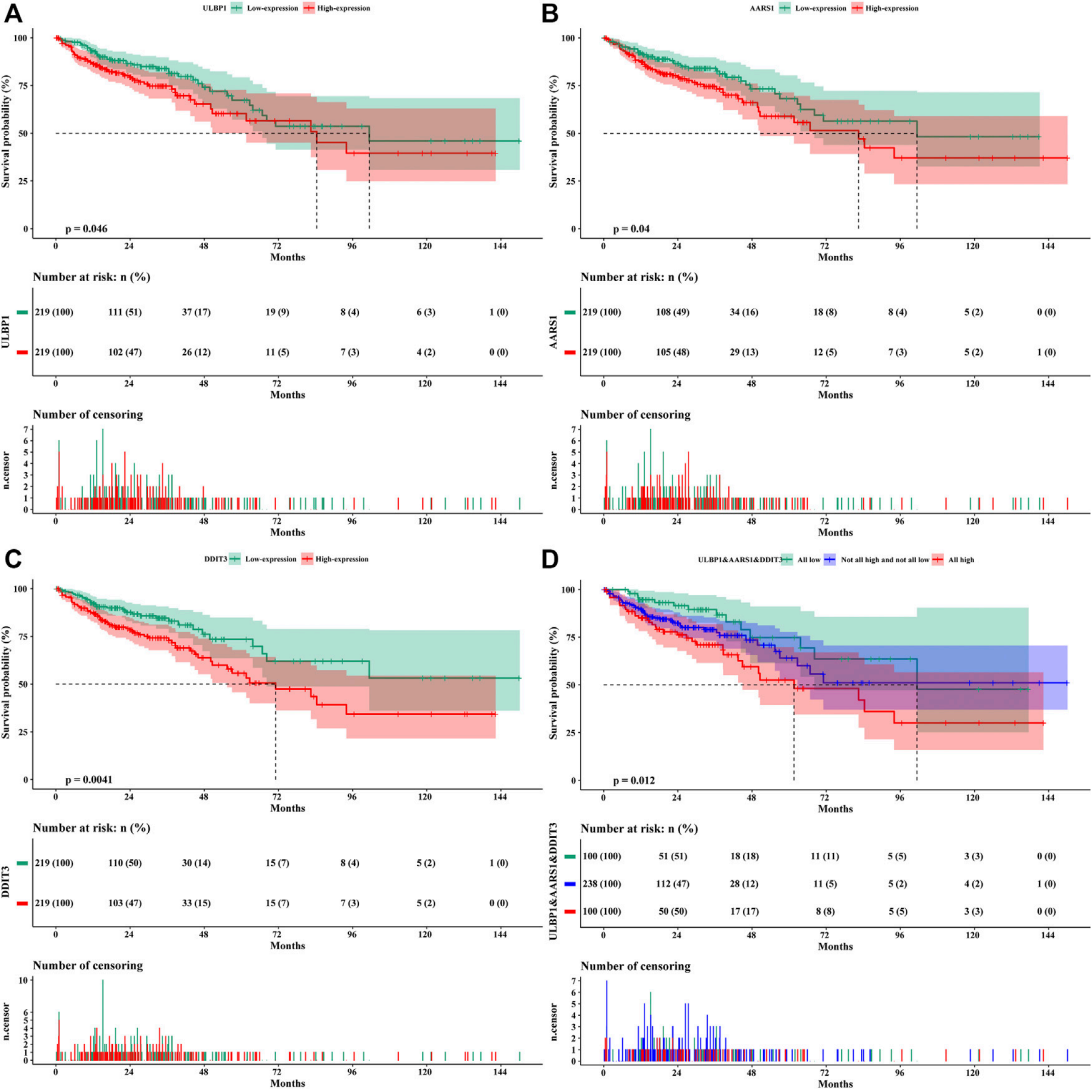
Kaplan–Meier survival curves of genes in COAD. **(A)** *ULBP1*; **(B)** *AARS1*; **(C)** *DDIT3*; **(D)** *ULBP1*& *AARS1* &*DD*IT3. Notes: COAD, colon adenocarcinoma; ULBP1, unique long 16 (UL16)-binding protein 1; AARS1, alanyl-tRNA synthetase 1; DDIT3, DNA damage inducible transcript 3.

The results of univariate survival analysis included the TNM stage as an adjusted prognostic factor. After adjustment, the high expression of *ULBP1* gene in COAD predicted a worse OS compared to patients with low expression of *ULBP1* (Adjusted HR = 1.544, 95%CI = 1.020–2.337, *p* = 0.040) (Figure 4A; Table 2).

**TABLE 2:** Prognostic values of *ULBP1* and top 20 *ULBP1*-coexpression genes in COAD

Gene	Patients	OS			
	(*n* = 438)	No. of events	MST (days)	HR (95%CI)	Adjusted *P* ^&^
*ULBP1*					
Low	219	43	3,042	1	0.040
High	219	55	2,532	1.544 (1.020–2.337)	—
*ZNF534*					
Low	219	47	3,042	1	0.645
High	219	51	2,475	0.908 (0.602–1.369)	—
*ZNF578*					
Low	219	47	2,821	1	0.144
High	219	51	2,532	1.357 (0.901–2.043)	—
*ZNF761*					
Low	219	42	3,042	1	0.382
High	219	56	2,532	1.373 (0.908–2.075)	—
*CLGN*					
Low	219	45	3,042	1	0.440
High	219	53	2,134	0.850 (0.563–1.284)	—
*ASNS*					
Low	219	46	2,134	1	0.405
High	219	52	2,821	1.191(0.789–1.798)	—
*TUBE1*					
Low	219	50	2,532	1	0.924
High	219	48	2,475	0.980(0.648–1.482)	—
*DMGDH*					
Low	219	47	3,042	1	0.810
High	219	51	2047	0.950 (0.623–1.448)	—
*UPK1A*					
Low	219	40	3,042	1	0.185
High	219	58	2,134	1.332 (0.872–2.037)	—
*DCDC1*					
Low	219	48	2,821	1	0.876
High	219	50	2047	1.033 (0.687–1.553)	—
*PSAT1*					
Low	219	48	2047	1	0.217
High	219	50	2,821	1.297 (0.858–1.961)	—
*AGBL3*					
Low	219	44	2,532	1	0.439
High	219	54	2,134	1.177 (0.779–1.777)	—
*SLC4A5*					
Low	219	47	3,042	1	0.848
High	219	51	2,532	0.960 (0.633–1.456)	—
*YARS*					
Low	219	47	2,475	1	0.545
High	219	51	3,042	1.135 (0.754–1.707)	—
*DDIT3*					
Low	219	35	NA	1	0.044
High	219	63	2,134	1.556 (1.013–2.390)	—
*AARS1*					
Low	219	41	3,042	1	0.031
High	219	57	2,475	1.583 (1.043–2.401)	—
*AGXT2*					
Low	219	45	2,475	1	0.372
High	219	53	2,821	1.208 (0.798–1.830)	—
*GARS*					
Low	219	49	2047	1	0.848
High	219	49	NA	0.961 (0.638–1.447)	—
*XPOT*					
Low	219	47	2,532	1	0.984
High	219	51	2,821	1.004 (0.666–1.513)	—
*NOL4*					
Low	219	46	NA	1	0.750
High	219	52	2,475	1.069 (0.710–1.609)	—
*PHGDH*					
Low	219	43	2,134	1	0.155
High	219	55	2,821	1.353 (0.892–2.054)	—

Notes: Adjusted *P* ^&^, adjustment for TNM stage; COAD, colon adenocarcinoma.

**FIGURE 5:**
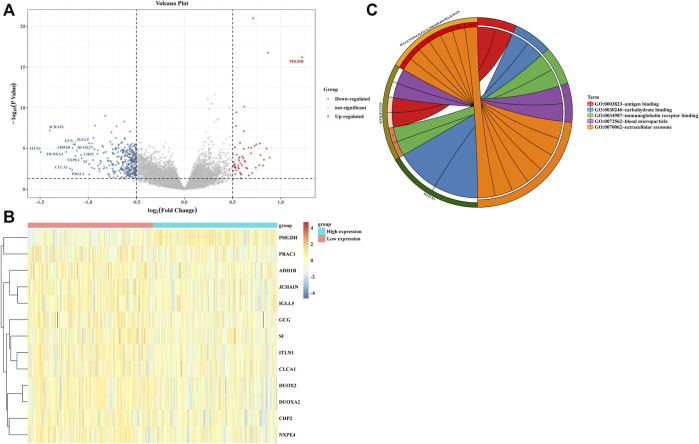
Difference and enrichment analysis of high- and low- expressed *ULBP1* groups in COAD. **(A,B)** differential expression analysis; **(C)** enrichment analysis. Notes: COAD, colon adenocarcinoma; *ULBP1*, unique long 16 (UL16)-binding protein 1.

We divided 438 COAD patients into high- and low-expression groups based on the median cut-off value of *ULBP1* expression. At the same time, the COAD genome-wide data was also divided into two groups, and the differential analysis and enrichment pathway analysis of these two groups were carried out. The *PHGDH* gene in the high expression group was significantly up-regulated, while the down-regulated genes included *ITLN1*, *JCHAIN*, *DUOXA2*, *CLCA1*, *PRAC1*, *ADH1B*, *GCG*, *IGLL5*, *NXPE4*, *DUOX2*, *CHP2*, and *SI*. The enrichment pathway analysis showed that these differential genes might involve in the process of extracellular exosome and immunoglobulin receptor binding (Figure 5).

**FIGURE 6:**
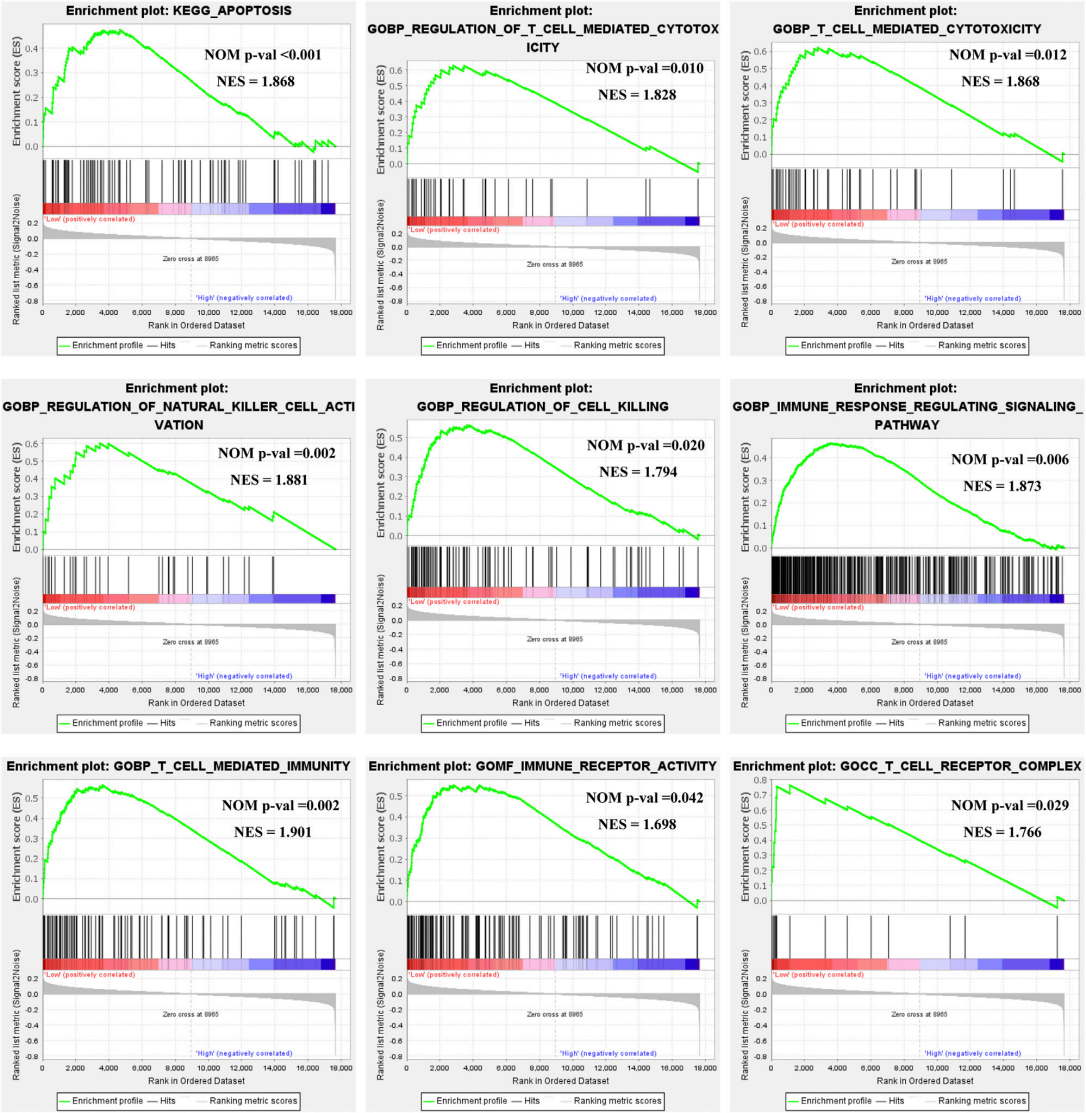
GSEA of *ULBP1* expression in COAD. Notes: COAD, colon adenocarcinoma; ULBP1, unique long 16 (UL16)-binding protein 1; GSEA, gene set enrichment analysis.

Based on the *ULBP1* expression levels, we performed the Gene Set Enrichment Analysis (GSEA) to investigate the potential prognosis molecular mechanism of *ULBP1* in COAD. The GSEA was performed by the tool of GSEA 4.1.0 version. The internal reference genes of GSEA were obtained from the Molecular Characterization Database (MSIGDB): KEGG pathway: c2.cp.kegg.v7.4.symbols.gmt; GO term: c5.go.v7.4.symbols.gm. In this study, nominal *p* < 0.05 and false discovery rate (FDR) < 0.25 were considered statistically significant. The results showed that *ULBP1* gene might involve in the development of COAD by participating in the apoptosis pathway and the biological process of T cell mediated cytotoxicity, regulation of natural killer cell activation, and T cell mediated immunity. (Figure 6; Supplementary Figure S1).

### UL-16 Binding Protein 1 Related Co-expression Analysis and Prognostic Analysis in Colon Adenocarcinoma

**FIGURE 7:**
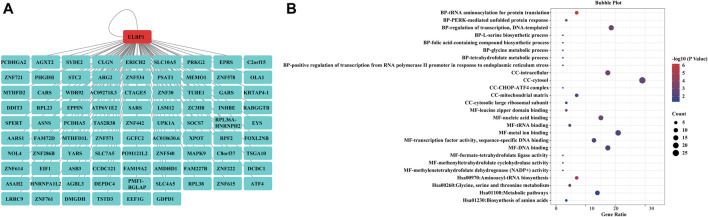
*ULBP1* related co-expressed genes and enrichment analysis in COAD. **(A)** *ULBP1* related co-expressed genes; **(B)** enrichmentanalysis. Notes: COAD, colon adenocarcinoma; *ULBP1*, unique long 16 (UL16)-binding protein 1.

Based on all gene expression sequences of the TCGA database, the potential clinical value of *ULBP1* gene and *ULBP1* related co-expressed genes was investigated. A total of 87 co-expressed genes related to *ULBP1* in COAD were mined. Pathway analysis of 87 co-expressed genes showed that co-expressed genes were involved in metabolic pathways of COAD (Figure 7).

The prognostic value of the top 20 *ULBP1*-related co-expressed genes in COAD has also been investigated. The multivariate survival analysis showed that patients with COAD with high expression of alanyl-tRNA synthetase 1 (*AARS1*) (Adjusted HR = 1.583, 95%CI = 1.043–2.401, *p* = 0.031) or DNA damage inducible transcript 3 (*DDIT3*) (Adjusted HR = 1.556, 95%CI = 1.013–2.390, *p* = 0.044) had worse OS when compared with patients with low expression of *DDIT3* or *AARS*, respectively (Figures 4B,C; Table 2). The combined analysis of *ULBP1*, *DDIT3*, and *AARS* genes showed that the risk of death in COAD patients with High *ULBP1* & High *DDIT3* & High *AARS1* was 2.210-fold higher than that of COAD patients with Low *ULBP1* & Low *DDIT3*& Low *AARS1* (Adjusted HR = 1.180–4.140, *p* = 0.013) (Figure 4D; Table 3).

**TABLE 3:** Combined effect survival analysis

Gene	Patients	OS			
	(*n* = 438)	HR (95%CI)	Crude P*	HR (95%CI)	Adjusted *P* ^&^
Low *ULBP1&* Low *DDIT3&* Low *AARS1*	100	1	—	1	—
Not all high or low	238	1.681 (0.940–3.004)	0.080	1.424 (0.778–2.606)	0.252
High *ULBP1&* High *DDIT3&* High *AARS1*	100	2.434 (1.325–4.470)	0.004	2.210 (1.180–4.140)	0.013

Notes: Adjusted *P* ^&^, adjustment for TNM stage; COAD, colon adenocarcinoma.

**FIGURE 8:**
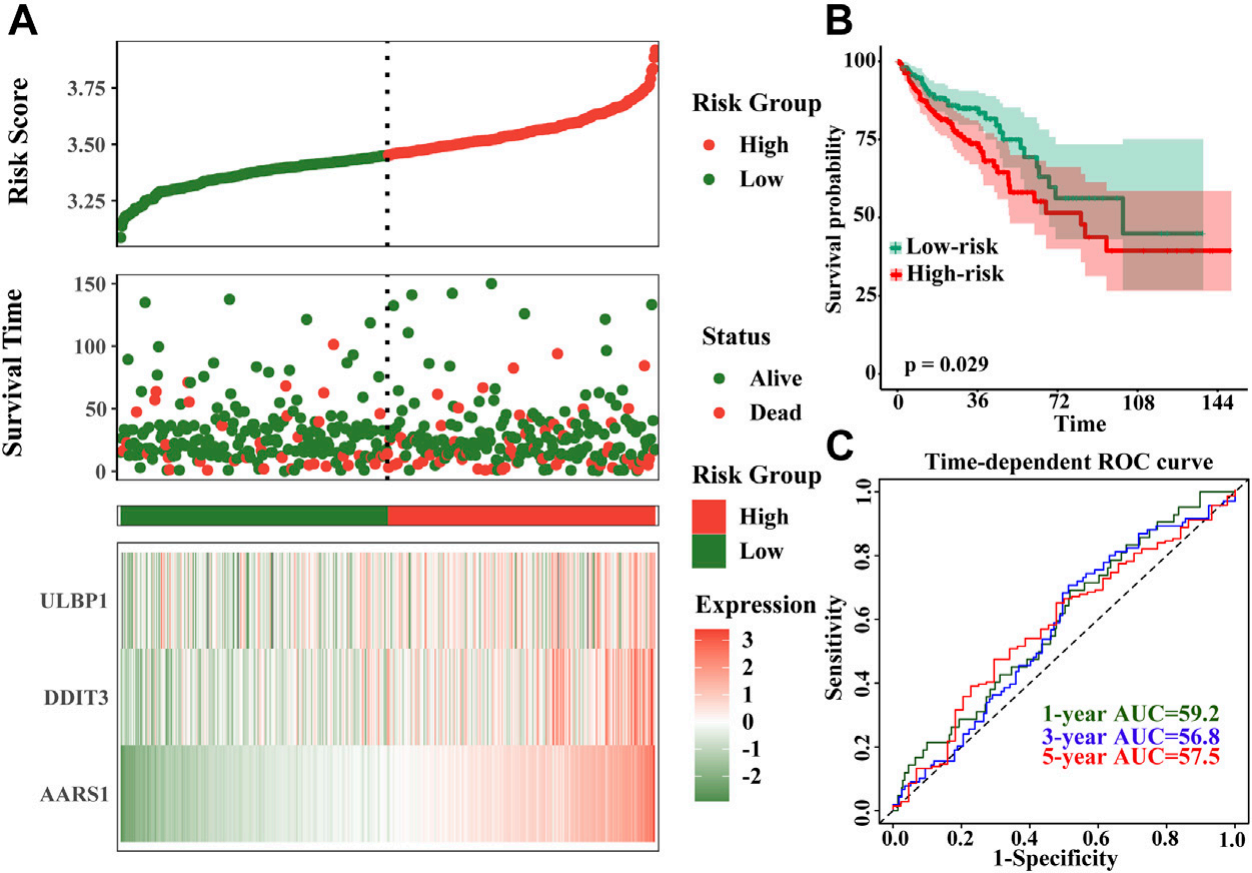
Prognostic risk score model and time-dependent ROC curve of *ULBP1* gene in COAD. **(A)** risk score developed by *ULBP1*, *DDITS*, and *AARS*; **(B)** kaplan–Meier survival curve of risk score; **(C)** time-dependent ROC curve. Notes: COAD, colon adenocarcinoma; ULBP1, unique long 16 (UL16)-bindingprotein 1; *AARS1*, alanyl-tRNA synthetase 1; *DDIT3*, DNA damage inducible transcript 3. ROC, receiver operating characteristic curve.

The model of risk score we constructed by the formula: Risk score = ULBP1*0.434 + DDIT3*0.442 + *AARS* *0.459. The higher the gene expression, the higher the risk score, and the higher the patient’s risk of death. The time-dependent ROC curve results showed that the 1-, 3-, and 5-year AUCs were 59.2, 56.8, and 57.5, respectively (Figure 8).

## Discussion

It is now widely accepted that tumors develop methods to evade anti-cancer immunity through a process called immunoediting (Dunn et al., 2004). Various evidence from *in vivo* models indicates that the immune system attacks early-stage tumors. Surviving cancer cells must adapt to avoid the immune system. This process is described as immunoediting, immune sculpting, or cancer immune evasion (Dunn et al., 2004). In recent years, studies on tumor models *in vivo* strongly indicate that the activated immune receptor NKG2D participates in the anti-cancer immune response, and it has also attracted much attention as a ligand of the NKG2D receptor (Smyth et al., 2005; Guerra et al., 2008; McGilvray et al., 2009). In humans, primary tumors and tumor cell lines express NKG2D ligands at a high frequency (McGilvray et al., 2009). As an important member of NKG2D ligand, *ULBP1* also plays an important role in immune regulation. The expression of *ULBP1* was associated with majority of immune cells, including NK cells, most NKT cells, *γδ* T^+^ and CD8 T+ cells. etc. Interestingly, we can take targeted chemotherapy based on the results of drug susceptibility to the immune-related *ULBP1* gene.

After mining through the database, it was found that *ULBP1* was highly expressed in the majority of tumors (including COAD), compared with adjacent tumor tissues. However, some tumors expressed the opposite trend, such as lung adenocarcinoma (LAUD) (Figure 2A). As previously described, it also showed that the expression of *ULBP1* in different cancers was different, but the general expression was frequently expressed in cancer tissues. When we analyzed the diagnostic value of *ULBP1* gene expression differences in COAD, whether it was the TCGA cohort or the Guangxi validated cohort, we found that *ULBP1* had a higher diagnostic value (TCGA cohort:0.959; Guangxi cohort:0.898) in COAD. In other words, we can take advantage of this high expression characteristic in cancer tissues, and the immune-related *ULBP1* can better distinguish cancer tissues from normal tissues. Additionally, we also found that with the progress of TMN staging, the expression of *ULBP1* showed an upward trend. The expression level of ULBP1 gene was related to the tumor grade and prognosis, and the differential expression level of ULBP1 gene was different in tumor tissues and adjacent normal tissues of patients with different tumor stages. Univariate and multivariate survival analysis results showed that low expression of *ULBP1* in patients with COAD had a worse prognosis when compared with those patients with high expression of *ULBP1*. The differential expression results between the high and low groups of *ULBP1* expression indicated that it was related to the binding of immunoglobulins. In addition, the GSEA of *ULBP1* gene in COAD suggested that *ULBP1* was involved in the occurrence and development of COAD through enrichment of apoptosis pathways, and was related to the immunoregulation of T cells and NK cells. We suspected that upregulated-*ULBP1* might participate in the apoptosis process of COAD through its unique immune regulation mechanism.

A study by CADOUX et al. also found that *ULBP1* was expressed at a higher level in hepatocellular carcinoma (HCC) tumors with lower differentiation and higher grades, but the difference is not significant (Cadoux et al., 2021). Interestingly, a study of 462 primary colorectal tumors by McGilvray et al. investigated the ULBP1-expressed protein in different TNM stages, the result showed the opposite trend was that high expression level of ULBP1 was common in TNM stage I tumors, but gradually decreased in stage II, III, and IV tumors (McGilvray et al., 2009). To understand the difference, we also investigated the expression level of *ULBP1* in rectal adenocarcinoma from the data platform (Supplementary Figure S2), the trend was consistent with the description of McGilvray et al. (McGilvray et al., 2009), indicating that the expression of *ULBP1* in the colon and rectum was also heterogeneous. Changes in the expression level of *ULBP1* are inseparable from tumor differentiation and grade. In other words, *ULBP1* is closely related to tumor prognosis. However, previous reports described the potential mechanism and prognosis of *ULBP1* expression changed. A study of genome-wide screen to identify novel drivers of *ULBP1* expression by Gowen et al. showed that in the multiple stages of *ULBP1* biogenesis, independent pathways gradually play a role. The transcription factor ATF4 drives the expression of *ULBP1* gene in cancer cells, while the RNA binding protein RBM4 supports the expression of *ULBP1* by inhibiting a new alternative splicing subtype of *ULBP1* mRNA, and explains its mechanism of activating the body’s immune system (Gowen et al., 2015). The study by Chava et al. indicated that DOT1L inhibition could regulate apoptotic and metabolic pathways as well as upregulate the expression of *ULBP1* that increased in NK cell-mediated ovarian cancer eradication (Chava et al., 2021). Maccalli et al. showed that patients with melanoma with the negative expression of sULBP-1 were associated with a better prognosis than those patients with positive expression of sULBP-1 (OS: 25.3 months *vs.* 12.1 months) (Maccalli et al., 2017). On the contrary, a study by CADOUX et al. showed that the high expression of *ULBP1* was related to the aggressiveness of hepatocellular carcinoma, and the expression of *ULBP1* could be down-regulated through the β-catenin signaling pathway (Cadoux et al., 2021). The study by McGilvray et al. also indicated that patients with ovarian cancer with high expression of *ULBP1* had a worse survival than those patients with no expression of *ULBP1* (disease-specific survival: 14 months *vs.* 30 months) (McGilvray et al., 2010).

The interaction between NKG2D and its ligands may play a central role in anti-tumor surveillance. The level of NKG2D ligands may determine the strength of the anti-tumor immune response (Wu et al., 2012). As described above, tumors can lead to tumor re-editing through immune evasion or ligand shedding. Different cancers are heterogeneous, and we should treat different cancers differently in their anticancer immune responses. To directly avoid NKG2D recognition, tumors may secrete TGF-*β* and/or release soluble NKG2D ligands, thereby down-regulating the expression of NKG2D (Lee et al., 2004). This observation was also observed in NKG2D knockout mice. For example, the incidence of MCA-induced fibrosarcoma was not affected when knocked out, but the incidence of large prostate tumors, when knocked out, was much higher than that of wild-type (Guerra et al., 2008). Butler et al. confirmed that p53 family members play an important role in the upregulation of *ULBP1* in head and neck squamous cell carcinoma induced by proteasome inhibitor drugs (Butler et al., 2009). It is well known that the activation of immune response by NKG2D depends on the tissue microenvironment and synergizes/antagonizes the signals induced by other cell receptors and cytokines (Eagle et al., 2009). A similar description was suggested by CADOUX et al. that the activated NKG2D system led to a strong inflammatory response, leading to a strong aggressiveness and poor prognosis (Cadoux et al., 2021). These factors vary for different types of cancer. It is also clear that NKG2D ligand can be independently expressed on cancer cells and can be expressed in response to different cancer-related pathways. Such as ULBP1-2, but not ULBP3, is induced by the expression of the BCR/ABL oncogene (McGilvray et al., 2010).

The enriched pathways of *ULBP1* gene and its co-expressed genes showed that co-expressed genes might participate in the metabolic pathway and Aminoacyl-tRNA biosynthesis of COAD. When we selected the top 20 co-expressed genes and performed prognostic analysis, we found that both ARRS1 and DDIT3 genes have prognostic value in COAD. Notably, the combination of High expression of ULBP1, AARS1, and DDIT3 would increase the 2.2-fold death risk of COAD, when compared with those of low expression genes. AARS1 is a family member of the aminoacyl-tRNA synthetases (AARSs), which is a housekeeping protein widely present in all organisms, it can catalyze the combination of amino acids and tRNA and convert nucleic acid coding information into amino acids, playing an important role in protein synthesis (Zhang et al., 2020). In addition to these translation functions, AARSs are also involved in many other important physiological activities, such as translation and transcription regulation, signal transduction, cell migration, angiogenesis, inflammation, and tumorigenesis (Kim et al., 2011; Datt and Sharma, 2014; Kim et al., 2014). Cancer is a disease of cell disorders, which can be affected by using translation in unexpected ways, using the catalytic function of AARSs in an untranslated environment, or manipulating its regulatory function independent of enzyme activity (Wang and Yang, 2020). If the expression of tRNA exceeds a certain level, it may cause abnormal cell and tissue growth. On the other hand, with the strong demand for protein synthesis by cancer, the classic enzyme action of AARSs is needed to maintain tumor growth (Grewal, 2015). DDIT3 gene, also called CHOP, is an endoplasmic reticulum (ER). This gene encodes a member of CCAAT/enhanced binding protein (C/EBP) family transcription factors (Ron and Habener, 1992). DDIT3, activated by p38 mitogen-related protein kinase, is a major pro-apoptotic transcription factor induced by ER stress (Woo et al., 2007). It has been reported that DDIT3 overexpression can lead to cell cycle arrest and/or apoptosis (Woo et al., 2007). Studies have also shown that DDIT3 can trigger key early events leading to cell apoptosis, which is considered an important target for the development of anti-cancer drugs (Oyadomari and Mori, 2004). Additionally, DDIT3 can participate in cell apoptosis transition and induce Bcl2 down-regulation and DR5 (death receptor 5) activated protein (Farooqi et al., 2015). RASK et al. also indicated that increased DDIT3 was associated with the tumor invasion of CRC (Rask et al., 2000). However, Sun et al. activated the PERK-ATF4-CHOP signaling pathway through TIIA, and then increased the expression of ULBP1 and DR5 through ATF4 and CHOP, leading to enhanced NK cell-mediated killing of NSCLC cells, which seemed to indicate a connection between ULBP1 and CHOP(Sun et al., 2021). In general, the combination of these 3 genes that reflect different levels can improve the prognosis of COAD patients.

However, our research still has some unavoidable limitations. Firstly, the study obtained fewer clinical parameters from the TCGA database, and more clinical parameters need to be included to reduce clinical bias. Secondly, we only analyze from the perspective of genes, but due to the limitations of the current experimental conditions, there is no protein-level validation. In the future, more experiments including *in vivo* and *in vitro* are needed to explore. Finally, the Guangxi cohort in this study is only a single-center cohort, and multiple centers and larger samples might be needed for further validation.

## Conclusion

Our study was the first to investigate the diagnostic and prognostic value of the immune-related *ULBP1* gene in COAD. *ULBP1* gene had a high diagnostic value in COAD. Up-regulated *ULBP1* gene of patients with COAD predicted a worse prognosis compared to those patients with down-regulated *ULBP1* gene. GSEA results showed that *ULBP1* was involved in the apoptotic pathway and biological process of T cell mediated cytotoxicity, regulation of natural killer cell activation, and T cell mediated immunity of COAD. The combination survival analysis showed that the combination of high expression of *ULBP1*, *AARS1*, and *DDIT3* would increase the 2.2-fold death risk of COAD when compared with those of low expression genes. However, these findings need to be further validated.

## Data Availability

The original contributions presented in the study are included in the article/Supplementary Material, further inquiries can be directed to the corresponding authors.

## Supplementary Material

Supplementary Figure S1 Apoptosis pathway.

Supplementary Figure S2 Expression of *ULBP1* in READ based on individual cancer stages.
