# Supplementary figures and images for: Immune ULBP1 is Elevated in Colon Adenocarcinoma and Predicts Prognosis
Source: Front Genet. 2022 Feb 8;13:762514. doi: 10.3389/fgene.2022.762514 (PMC8862730; doi:10.3389/fgene.2022.762514)

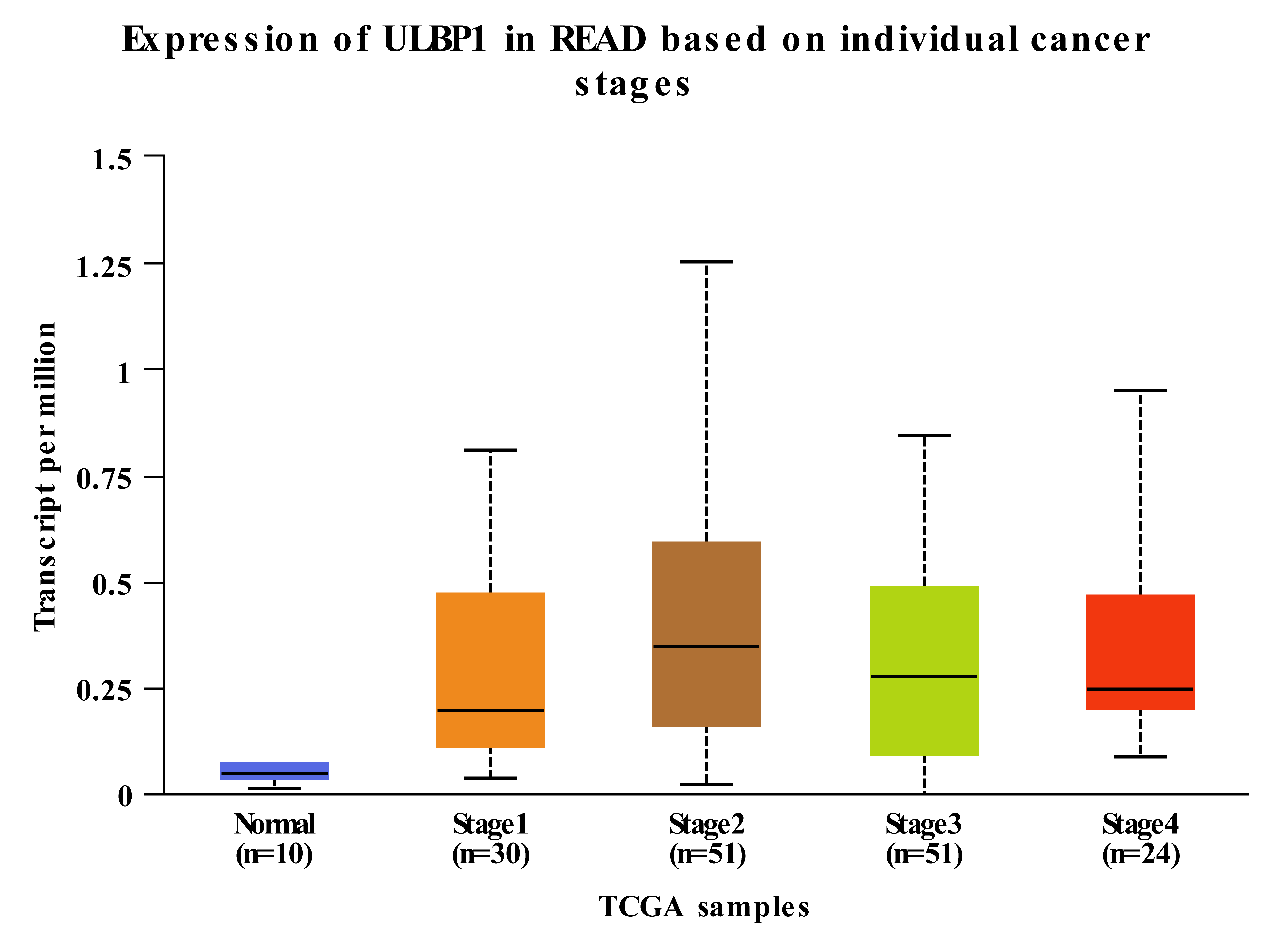

Supplement: Supplementary file 1 [file Image2.TIF]
